# A patient presenting with acute abdomen due to metastatic small bowel melanoma: a case report

**DOI:** 10.1186/1752-1947-7-216

**Published:** 2013-08-23

**Authors:** Georgios D Lianos, Thomas Messinis, Rodamanthos Doumos, Alexandra Papoudou-Bai, Christina D Bali

**Affiliations:** 1 Department of Surgery, University Hospital of Ioannina, St Niarchou Av. Ioannina, 45110, Greece; 2 Department of Pathology, University Hospital of Ioannina, St Niarchou Av., Ioannina 45110, Greece

**Keywords:** Acute abdomen, Melanoma, Metastasis, Perforation

## Abstract

**Introduction:**

Malignant tumors of the small bowel are rare. Melanoma of the small intestine is in most cases metastatic from a primary skin lesion. Perforation of small bowel melanoma is an extremely rare entity. To the best of our knowledge this is the fifth case published to date.

**Case presentation:**

We report a rare case of acute abdomen due to perforated metastatic small bowel melanoma in a 38-year-old Caucasian man.

**Conclusions:**

In the majority of cases small bowel melanoma represents metastasis from cutaneous sites. Although rare, the possibility of abdominal metastatic melanoma presenting with the clinical picture of acute abdomen must be always considered by the operating surgeon in patients with a history of primary cutaneous malignant lesion.

## Introduction

Small bowel melanoma is rare and often asymptomatic. Most common symptoms are chronic abdominal pain, bleeding and intestinal obstruction or intussusception [1]. Rarely, a small bowel melanoma occurs with the clinical picture of acute abdomen due to perforation [2]. Correct diagnosis is difficult and most often it can be obtained only after explorative laparotomy. To date, only a few reports of small bowel perforation due to metastatic melanoma have been published [3-5]. We share our experience of successful treatment of such a rare entity.

## Case presentation

**Figure 1:**
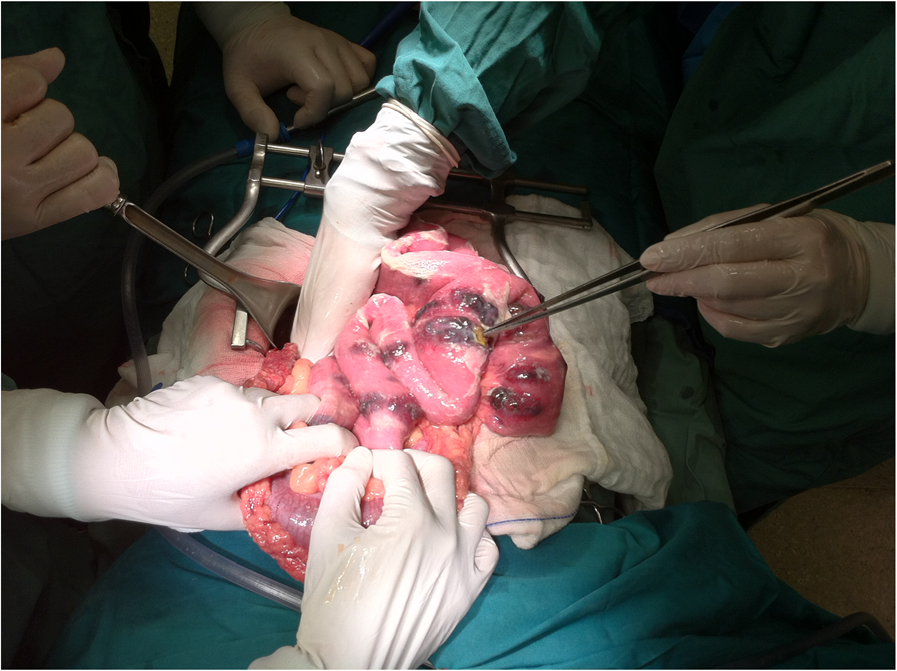
Multiple metastatic melanoma nodules of the jejunum, one of which has been perforated.

**Figure 2:**
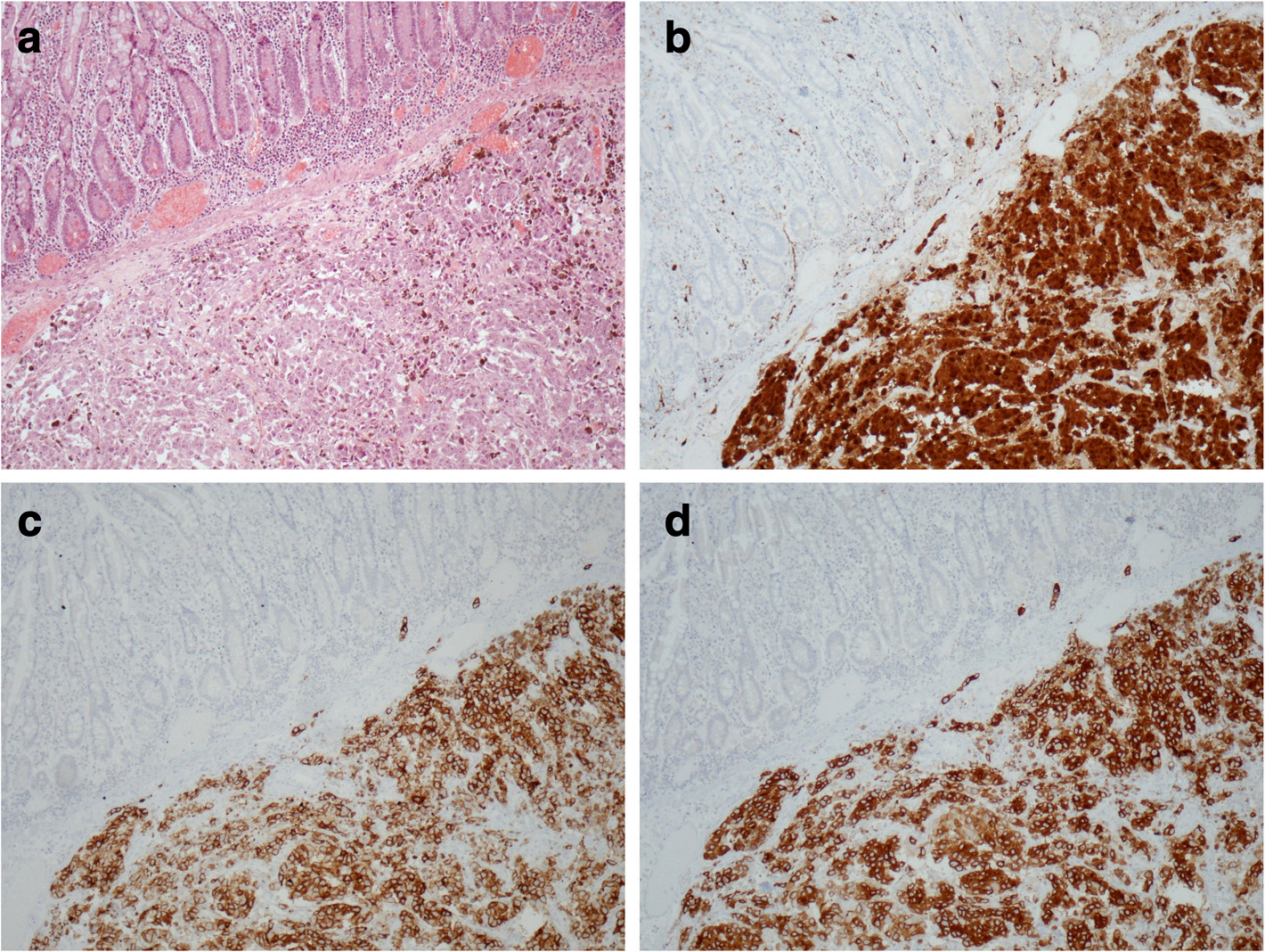
(a) Invasion of the intestinal wall from marked atypical neoplastic cells containing melanin pigmentation (hematoxylin and eosin ×100); (b) immunohistochemically, tumor cells were reactive for S-100 protein, (c) HMB-45, and (d) melan-A (3,3'-diaminobenzidine ×100).

A 38-year-old Caucasian man came to the emergency department of our hospital with diffuse abdominal pain for the past 10 hours. A few months ago he was diagnosed with metastatic brain melanoma and he has been treated since then with high doses of corticosteroids. At follow up, 20 days before admission, he had computed tomography (CT) scans of his brain, chest and abdomen showing only the known brain lesions. At that time, his vital signs showed only mild tachycardia. On physical examination abdominal sounds were absent and his abdomen was rigid and diffusely tender with rebound tenderness. A rectal examination showed an empty rectum. The emergent laboratory tests revealed as follows: white blood cells 10,200mm^3^, hemoglobin at 11.5g/dL, c-reactive protein was at 14mg/L, the results of creatinine, electrolytes, and liver function tests were normal. The admission chest X-ray showed pneumoperitoneum. An exploratory laparotomy was performed under general anesthesia on the same day. Intraoperative findings revealed multiple (>100) black-colored nodules, of different size (0.5 to 7.0cm) on the small bowel wall as well as on the peritoneum surface. One of the nodules located in his jejunum was perforated (Figure 1). A long part of his small bowel (approximately 2m), containing most of the nodules, was resected for cytoreduction purposes and also to prevent another perforation in the near future. The postoperative period was uneventful and the patient was discharged 9 days later. Six months after the operation the patient remains asymptomatic. The histopathological diagnosis was malignant melanoma of the small bowel. There was diffuse infiltration of the intestinal wall from atypical large neoplastic cells with abundant eosinophilic cytoplasm. Melanin pigmentation was observed in the neoplastic cells (Figure 2a). Immunohistochemistry showed that the tumor cells were positive for S-100 protein (Figure 2b), HMB-45 (Figure 2c), melan A (Figure 2d) and vimentin, whereas they were negative for CD45-common leukocyte antigen and pancytokeratin.

## Discussion

Small bowel melanoma is a rare entity with difficult diagnosis. The majority of these neoplastic lesions are metastatic from primary skin lesions [6]. It seems that a clear histological distinction between primary small bowel and metastatic intestinal melanoma is very difficult to establish. In most cases, metastatic melanoma of the small bowel is clinically silent. An acute presentation may rarely occur due to intestinal obstruction or intussusception. The clinical picture of acute abdomen due to perforation of small bowel metastatic melanoma is extremely rare. To date only five cases, including our own, of perforation due to melanoma intestinal metastasis have been reported [1,7,8].

Preoperative diagnosis of small bowel melanoma is difficult to establish due to the non-specific clinical symptoms. Imaging methods such as capsule endoscopy, CT, magnetic resonance imaging (MRI) and positron emission tomography (PET) scan may generate a suspicion of small bowel melanoma. It has been reported that capsule endoscopy is sensitive in detecting small bowel metastasis, whereas extra-intestinal intra-abdominal involvement might be detected by MRI, CT and PET scan [8]. Definitive diagnosis of small bowel metastasis can be obtained only after explorative laparotomy and bowel resection as an elective method or in an emergency setting, as in our case, and may improve the overall prognosis of the patient [9,10].

## Conclusions

Abdominal metastatic melanoma rarely presents as an acute abdomen. Intestinal metastatic melanoma should be considered in the differential diagnosis of acute abdomen in patients with a history of primary cutaneous melanoma.

## Consent

Written informed consent was obtained from the patient for publication of this case report and accompanying images. A copy of the written consent is available for review by the Editor-in-Chief of this journal.

## Competing interests

The authors declare that they have no competing interests.

## Authors’ contributions

GL analyzed and interpreted the patient data, was actively involved in the operation and drafting the manuscript. AP was the corresponding pathologist and contributed in writing the manuscript. TM, RD and CB performed the operation and have contributed in writing the manuscript. All authors read and approved the final manuscript.
